# Drug-drug interactions in older patients with cancer: a report from the 15th Conference of the International Society of Geriatric Oncology, Prague, Czech Republic, November 2015

**DOI:** 10.3332/ecancer.2016.611

**Published:** 2016-01-14

**Authors:** Rob Stepney, Stuart M Lichtman, Romano Danesi

**Affiliations:** 1 2 Walcot Farm Cottages, Charlbury, Oxfordshire, OX7 3HJ, UK; 2 Memorial Sloan Kettering Cancer Center, Commack, NY, USA; 3 Department of Clinical and Experimental Medicine, University of Pisa, Italy

**Keywords:** elderly, cancer, polypharmacy, drug interaction, adverse drug events, tyrosine kinase inhibitors

## Abstract

Drugs taken for cancer can interact with each other, with agents taken as part of supportive care, with drugs taken for comorbid conditions (which are particularly common in the elderly patients), and with herbal supplements and complementary medicines. We tend to focus on the narrow therapeutic window of cytotoxics, but the metabolism of tyrosine kinase inhibitors by the cytochrome P450 3A4 enzyme (CYP3A4) makes some TKIs particularly prone to interference with or from other agents sharing this pathway. There is also potential for adverse pharmacokinetic interactions with new hormonal agents used in advanced prostate cancer.

## Introduction

Given the potential for drug-drug interactions (DDIs) in elderly cancer patients, it was appropriate and helpful for the recent 15th Conference of the International Society of Geriatric Oncology (SIOG), held in Prague, Czech Republic in November 2015 to devote a plenary session to this topic. It was chaired by Stuart Lichtman (Memorial Sloan Kettering Cancer Centre, Commack, New York, USA) and Romano Danesi (Department of Clinical and Experimental Medicine, University of Pisa, Italy).

## Summary of presentations

Recent registry data covering 310,000 people in Scotland show that polypharmacy in general is a growing problem, and one highly correlated with increasing age [1]. Around 13% of adults were judged to be at risk of a serious DDI, and the single most important risk factor was the number of drugs dispensed. Compared with taking two to four drugs, taking five to nine increased the DDI risk four fold, and taking ten to fourteen drugs increased it by a factor of 12.

Among older cancer patients, polypharmacy is the norm, Holly Holmes (University of Texas Health Science Centre, Houston, USA) told the Prague conference. Data from the United States show that 40% of patients aged 65 and above take five or more different medicines, and 20% take ten or more [2]. In addition, 49% take at least one supplement.

Typically, people with cancer have two or three comorbidities, each requiring two or three drugs. Cancer and ageing coincide as causes of polypharmacy. The potential for pharmacokinetic (PK) and pharmacodynamic (PD) interactions is therefore large, as is the risk of adverse consequences, particularly since anticancer drugs tend to have a narrow therapeutic window.

According to an overview of published studies, common cancer-related drug interactions include phenytoin reducing the efficacy of irinotecan by inducing its hepatic metabolism, the potentiation of cyclophosphamide and fluorouracil-related neutropenia by thiazide diuretics, and the inhibition of warfarin metabolism, by drugs as various as capecitabine, carboplatin, and paclitaxel [3].

In a retrospective study in patients aged 70 or older having chemotherapy, potential drug interactions were identified in 75% [4]. Drug interactions were classified according to five levels, and at the most serious level almost doubled the risk of severe non-haematological toxicity. Substantial risk of DDIs is also present when consideration is restricted to ambulatory patients on oral anticancer agents [5]. Van Leeuwen *et al* found potential DDIs in 46% of outpatients treated at three Dutch centres. In 16% there was potential for a major DDI. These risks most frequently involved coumarins and opioids. The authors were particularly concerned about the CNS interactions that might lead to falls, drug combinations that have potential to prolong the QT interval, and those that can cause gastrointestinal toxicity.

Tyrosine kinase inhibitors (TKIs) are not exempt from potential adverse drug interactions [6]. The absorption of epidermal growth factor receptor (EGFR) TKIs, for example, is affected by gastric pH—and hence by concomitant use of antacids, H2 antagonists, or proton pump inhibitors. Gefitinib and erlotinib are metabolised by CYP enzymes. Hence concomitant treatment with the CYP3A4 inhibitor ketoconazole, for example, increases the area under the curve (AUC) and could result in increased toxicities such as skin rash or diarrhoea. The relative contribution of each CYP enzyme differs between TKIs, but there is wide potential for interaction with drugs used in supportive therapy or for comorbidities and those which involve these pathways.

When all DDIs are aggregated, they account for 20–30% of all adverse drug events, as estimated by Scripture and Figg [7]. Stuart Lichtman (Memorial Sloan Kettering Cancer Centre, Commack, USA) drew particular attention to the following possibilities: altered coagulation in patients taking warfarin and capecitabine, increased exposure to the active metabolite of irinotecan when taken together with ketoconazole, and increased clearance of imatinib in patients taking St John’s wort, which is an inducer of CYP3A4. Herbal supplements such as echinacea, kava, grape seed and Hypericum perforatum are also thought to be enzyme inducers.

In castrate-resistant prostate cancer, the androgen receptor remains a target, meaning that most patients will be treated with androgen deprivation therapy. In the pivotal trials, both abiraterone and enzalutamide improved survival in men over the age of 65 years. But both these agents have metabolic profiles that incur risk of DDIs [8, 9]. These were considered by William Dale, University of Chicago, USA, and Romano Danesi, Department of Clinical and Experimental Medicine, University of Pisa, Italy.

Since abiraterone is metabolised by CYP3A4, its plasma level can be increased by strong inhibitors of this enzyme such as ketoconazole, itraconazole, and verapamil. Abiraterone inhibits CYP2C8 and 2D6 and hence may increase plasma levels of substrates of these enzymes. Substrates of CYP2D6 include amitriptyline, oxycodone, and risperidone; and substrates of 2C8 include amiodarone and carbamazepine For these and other agents, therapeutic drug monitoring is recommended.

Enzalutamide is a moderate inducer of CYP2C9 and 2C19 and a strong inducer of CYP3A4. Plasma levels of substrates of these enzymes may be reduced when taken concomitantly. The effect on CYP3A4 may be clinically relevant as up to 60% of all drugs are metabolised via CYP3A4. Enzalutamide is extensively metabolized by CYP2C8. If coadministered with strong CYP2C8 inhibitors such as montelukast, trimethoprim, gemfibrozil, or pioglitazone, plasma levels are likely to be raised. Strong inducers of CYP2C8 may reduce the effectiveness of enzalutamide and hence should be avoided.

Growing awareness of the importance of DDIs in cancer patients is now reflected in the variety of sources offering information and guidance. These were described at the SIOG conference by Vincent Launay-Vacher of the Pitié-Salpêtrière University Hospital, Paris, France. Resources include the summaries of product characteristics, which give useful background information but address regulatory rather than practical issues. More helpful are sites provided by professional organisations such as the European Society of Medical Oncology (ESMO). ESMO has developed an on-line resource available at oncologypro.esmo.org, though only to health care professionals who are members of the Society [7]. This provides an overview of the main types of DDI with the eight most frequently used TKIs, their prophylaxis and treatment, and information (which can be downloaded) for patients.

The www.drugs.com site has a drug interactions checker providing information for health professionals as well as for patients. Both are freely accessible. The site allows the different agents in a prescription to be analysed at the same time and provides data on drug-food interactions, but is probably not exhaustive in its coverage of DDIs. The website SiteGPR provides evidence-based advice on dose adjustment in patients with renal impairment, including those that may be required because of DDIs.

## Conclusion

Dr Lichtman drew the following broad conclusions. Elderly patients take more medications than any other age group. Age-related changes in physiology and drug handling, plus comorbidities and associated medications, result in altered pharmacokinetics and pharmacodynamics. Adverse drug reactions are common and their risk increases with the number of drugs used. Nonprescription and herbal therapies are frequently taken and are of concern as demonstration of safety is not required prior to marketing. Also, there is no standardisation of manufacturing; and the fact that there is no requirement for ingredients to be clearly stated on the packaging raises a cause for concern, if not alarm, about their potential to cause unanticipated DDIs.
